# Impedance Spectroscopy Study of the Effect of Environmental Conditions on the Microstructure Development of Sustainable Fly Ash Cement Mortars

**DOI:** 10.3390/ma10101130

**Published:** 2017-09-25

**Authors:** José Marcos Ortega, Isidro Sánchez, Miguel Ángel Climent

**Affiliations:** Departamento de Ingeniería Civil, Universidad de Alicante, Ap. Correos 99, 03080 Alacant/Alicante, Spain; isidro.sanchez@ua.es (I.S.); ma.climent@ua.es (M.Á.C.)

**Keywords:** fly ash, microstructure, impedance spectroscopy, environmental conditions, temperature, relative humidity, sustainability

## Abstract

Today, the characterisation of the microstructure of cement-based materials using non-destructive techniques has become an important topic of study, and among them, the impedance spectroscopy has recently experienced great progress. In this research, mortars with two different contents of fly ash were exposed to four different constant temperature and relative humidity environments during a 180-day period. The evolution of their microstructure was studied using impedance spectroscopy, whose results were contrasted with mercury intrusion porosimetry. The hardening environment has an influence on the microstructure of fly ash cement mortars. On one hand, the impedance resistances R_1_ and R_2_ are more influenced by the drying of the materials than by microstructure development, so they are not suitable for following the evolution of the porous network under non-optimum conditions. On the other hand, the impedance spectroscopy capacitances C_1_ and C_2_ allow studying the microstructure development of fly ash cement mortars exposed to those conditions, and their results are in accordance with mercury intrusion porosimetry ones. Finally, it has been observed that the combined analysis of the abovementioned capacitances could be very useful for studying shrinkage processes in cement-based materials kept in low relative humidity environments.

## 1. Introduction

Firstly, the use of active additions in cement industry has been an important research field [1,2,3,4,5,6,7,8,9,10], because they provide very important economic and ecological benefits, like the reduction of CO_2_ emissions and the reuse of wastes coming from other industries. The particular case of fly ash and its effect on the properties of cement-based materials has been the topic of many studies [11,12,13]. This type of addition reacts with portlandite formed during the clinker hydration, producing new hydrated products which improve the properties of mortars and concretes [12,13,14,15,16,17]. Many studies show that cementitious materials with fly ash have very good durability properties in laboratory conditions [11,18], such as their permeability [19] and resistance to aggressive ion ingress [20,21,22,23]. This good durability is a consequence of the effects produced by fly ash in the microstructure of cement-based materials. This addition makes a denser pore structure of concrete at later ages [11,14,24] due to the development of pozzolanic reactions of fly ash, so an important pore refinement is produced [22,25], which improves the service properties of concretes and mortars [26]. This fact reveals the importance of characterising the microstructure of these materials, especially in studies in which cements with active additions are involved.

Nowadays, the study of the microstructure of cementitious materials using non-destructive techniques, such as shear wave velocity, SEM/ESEM, and thermal conductivity tests, has become a major topic of study [27,28,29,30,31,32,33]. Among them, the impedance spectroscopy has recently experienced great progress. This technique is based on the idea of correlating dielectric and mechanical properties of a solid material [34,35] and it has many advantages compared to other classical techniques, like mercury intrusion porosimetry. For example, it permits obtaining global information of the microstructure of the sample and to follow their evolution of the same one during the study period. Impedance spectroscopy has been mainly used for studying the pore structure of ordinary Portland cement-based materials [36,37], although several researches [22,38,39] have recently been published in which the microstructure evolution of samples with active additions has been followed with this technique.

Real structures are usually exposed to different hardening conditions depending on their geographical location. The different environmental temperatures and relative humidities can affect the development of clinker hydration [38,40,41,42]. As has been previously explained, the portlandite produced by this hydration is essential to initiate the fly ash pozzolanic reactions. As a consequence, the temperature and relative humidity would also affect the microstructure development of cement-based materials with fly ash, although the influence of these parameters has only been studied separately and until relatively recently, early hardening ages [40,42,43,44,45].

On the other hand, regarding the microstructure characterisation using impedance spectroscopy of cement-based materials with active additions, hardened in non-optimal conditions, there is only a previous recent authors’ work [38] in which ordinary Portland cement (OPC) and slag cement mortars were studied. Thus, the present research continues that previously mentioned work [38], but now fly ash cements has been studied. Therefore, the objective of this work is to study the influence of the combined relative humidity and temperature environmental conditions on the microstructure, especially the solid phase, of fly ash cement mortars in the long-term, using impedance spectroscopy, and mercury intrusion porosimetry as a contrasting technique.

## 2. Materials and Methods

### 2.1. Sample Preparation

Cement mortars were studied in this research. They were prepared using two different commercial fly ash cements, a Portland cement with fly ash, CEM II/B-V 42.5 R [46] (CEM II hereafter), which has a content of fly ash of between 21% and 35%, and a pozzolanic cement, CEM IV/B(V) 32.5 N [46] (CEM IV hereafter), whose content of fly ash was from 36% to 55% of the total binder.

Two different water to cement ratios, 0.4 and 0.5, were used. The aggregate to cement ratio was 3:1 for all the mortars. Cylindrical specimens of 10 cm diameter and 15 cm height were made. They were maintained in 95% RH chamber and 20 °C for 24 h. Following this period, they were de-moulded and cut into disks of approximately 1 cm thickness.

### 2.2. Environmental Conditions

Four different conditions were studied (see Table 1). The first one (condition A) was an optimum laboratory condition of 20 °C and 100% relative humidity (RH). Conditions B (15 °C and 85% RH) and C (20 °C and 65% RH) are representative of Atlantic and Mediterranean climates, respectively, which are present in different areas of the Iberian Peninsula (Spain and Portugal). The temperature and RH of both conditions represent the annual average values for each climate. Finally, a more extreme condition was studied, at 30 °C and 40% RH, called condition D.

For achieving the abovementioned conditions, the mortar samples were introduced into hermetically closed containers [38], which contained water or glycerol solutions, prepared according to the standard DIN 50 008 part 1 [47]. Finally, the containers were kept into chambers with controlled temperatures.

### 2.3. Mercury Intrusion Porosimetry (MIP)

For checking the impedance spectroscopy results, the microstructure of the mortars was also studied using mercury intrusion porosimetry [48,49,50,51]. The tests were performed with a Micromeritics Autopore IV 9500 porosimeter (Norcross, GA, USA). Before the test, samples were oven dried for 24 h at 105 °C. For each age, two measurements were performed on each material. Total porosity, pore size distribution [52], and percentage of Hg retained at the end of the experiment [36] were studied. The testing ages were 7, 28, 90, and 180 days.

### 2.4. Impedance Spectroscopy (IS)

**Figure 1:**
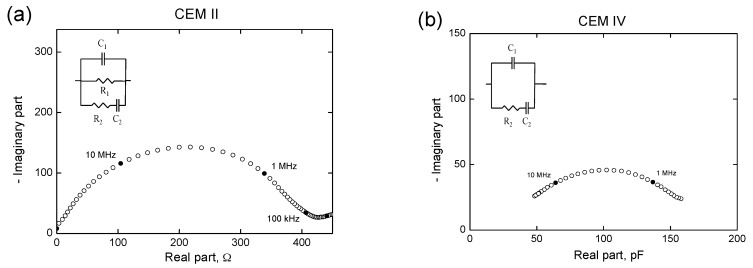
(**a**) Nyquist plot obtained for a CEM II (w:c ratio 0.4) exposed to condition A during 93 days, and the equivalent circuit used for fitting the measurements with the contacting method; (**b**) Cole-Cole plot for a CEM IV mortar (w:c ratio 0.4) at 24 days of hardening under condition A, and the equivalent circuit used for fitting the measurements with the non-contacting method.

The impedance measurements were performed using an Agilent 4294A analyzer (Agilent Technologies, Kobe, Japan) [38]. The measurements were taken over a frequency range of 100 Hz to 100 MHz. The electrodes used were circular (Ø = 8 cm) and made of flexible graphite, attached to a copper piece with the same diameter. Both contacting and non-contacting methods were used [36,38]. The measured data were fitted to the equivalent circuits proposed by Cabeza et al. [36] (see Figure 1), which include two time constants.

**Figure 2:**
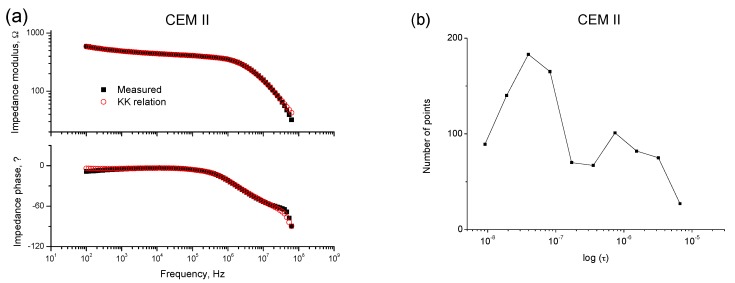
(**a**) Example of a Bode plot obtained for a CEM II mortar (w:c ratio 0.4) exposed to condition A for 93 days, and validated using the Kramers-Kronig (K-K) relations; (**b**) Differential impedance analysis of impedance spectrum shown in (**a**).

**Figure 3:**
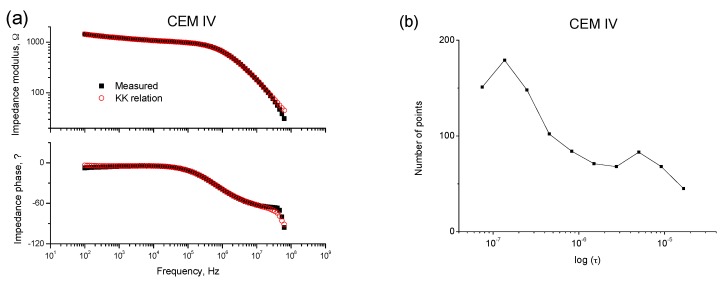
(**a**) Example of a Bode plot obtained for a CEM IV mortar (w:c ratio 0.4) exposed to condition A for 65 days, and validated using the K-K relations; (**b**) Differential impedance analysis of impedance spectrum shown in (**a**).

The causal, linear, and stable nature of the data recorded was validated using the Kramers-Kronig (K-K) relations [53] (see Figure 2a and Figure 3a). Moreover, the differential impedance analysis [36] was made on the spectra before assuming the equivalent circuit was valid for fly ash mortars. The two maxima observed in Figure 2b and Figure 3b reveal the presence of two-time constants in both impedance spectra, so this result indicates that the abovementioned equivalent circuits can be used for fly ash cement mortars.

For each cement type, condition and w:c ratio four different samples were tested. The evolution of impedance parameters with time is reported to over a 180 hardening day period.

## 3. Results and Discussion

### 3.1. Mercury Intrusion Porosimetry

**Figure 4:**
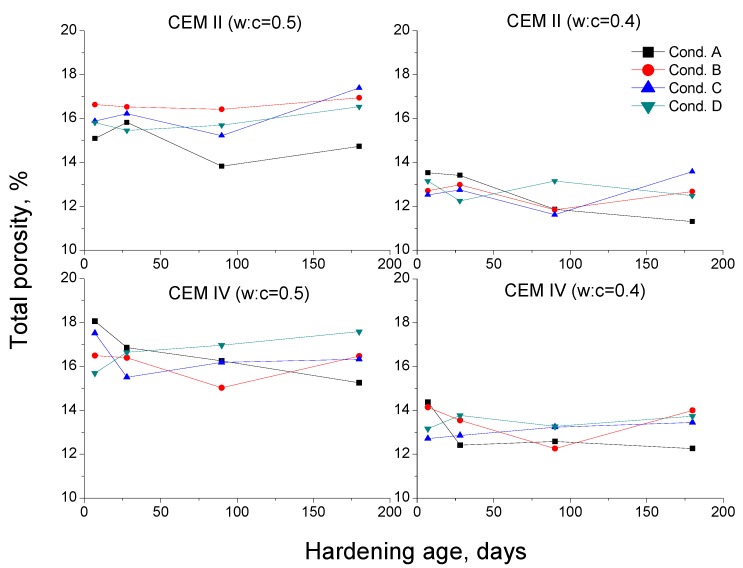
Results of total porosity for CEM II and IV mortars.

**Figure 5:**
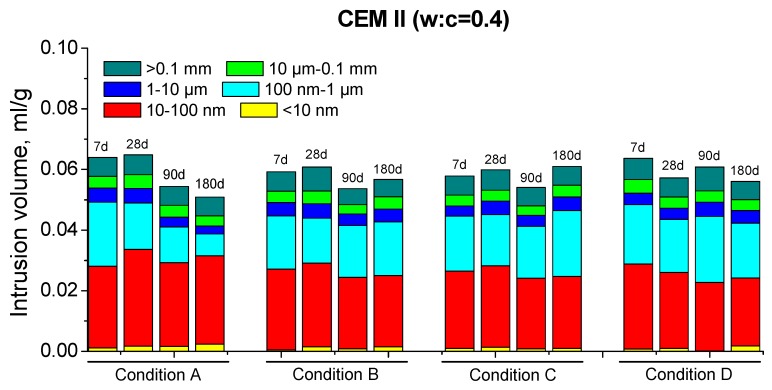
Pore size distributions for CEM II mortars with w:c ratio 0.4.

**Figure 6:**
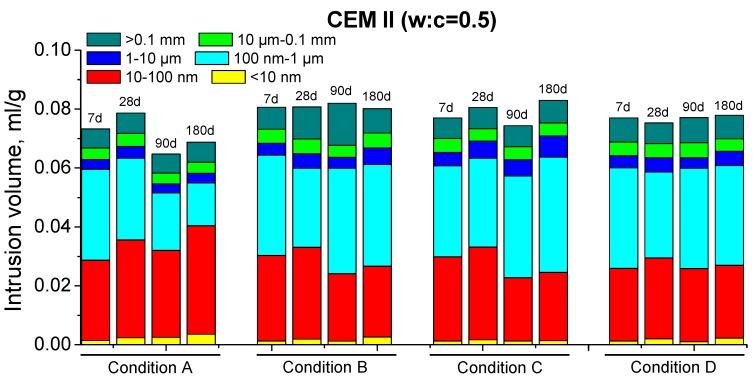
Pore size distributions for CEM II mortars with w:c ratio 0.5.

The results of total porosity for CEM II mortars are depicted in Figure 4. Their pore size distributions are shown in Figure 5 (w:c ratio 0.4) and Figure 6 (w:c ratio 0.5).

For those mortars, the high total porosity decrease and the progressive pore network refinement with time observed for condition A could be related to the very high RH present in the environment, combined with a high enough temperature. The unlimited availability of water would make easier the development of clinker hydration [38,39], so more portlandite would be formed at the early ages. Then, the pozzolanic reactions of fly ash could begin sooner [12,13,15], leading to a more compact microstructure [11,22].

The slower decrease of porosity (Figure 4) and the lower pore network refinement (Figure 5 and Figure 6) observed for CEM II mortars hardened in condition B could be due to the lower temperature of this environment. The clinker hydration is slowed down in environments with low temperatures [38,54], so more time would be needed for having enough portlandite available for starting the fly ash pozzolanic reactions. Moreover, the lower temperature would slow down these reactions [42]. As a consequence, the solid formation as products of pozzolanic reactions is also slower, which could explain the scarce fall with age of CEM II total porosity. On the other hand, the relatively high RH in condition B would provide enough water, which would allow the development of hydration and pozzolanic reactions in the long-term. However, it seems that CEM II samples exposed to condition B would need hardening times longer than 180 days for reaching similar porosities than those observed for condition A. The practically no decrease of total porosity of CEM II mortars with w:c ratio 0.5, compared to 0.4 ones, would suggest that the first ones would be more affected by environment B.

The similar values of total porosity obtained for conditions A and C in the short-term (see Figure 4) could be related to the high enough temperature of condition C. This shows that the clinker hydration reactions for condition C were developed with a similar velocity than for condition A, in spite of the lower RH [38,55]. This circumstance would entail that the fly ash pozzolanic reactions begin promptly, as suggested by the higher microstructure refinement observed for condition C at 28 days, compared to the rest of non-optimal environments (see Figure 5 and Figure 6). Nevertheless, in the long-term, the microstructure hardly improved. This would be due to the effect of the low RH of this environment, which would complicate the continuity of hydration [38] and pozzolanic reactions. In addition to this, the rise of total porosity at later ages and the loss of pore refinement would suggest the possible formation of shrinkage microcracks in the CEM II samples, caused by the water shortage in the environment [38,56].

The lower total porosities (see Figure 4) and the refined microstructure (see Figure 5 and Figure 6) showed by the CEM II mortars exposed to condition D at early ages could be related to the high temperature of this environment, which would accelerate the clinker hydration [38,40,42]. This fact would bring about a quick formation of portlandite, which would permit a relatively early beginning of fly ash pozzolanic reactions. Furthermore, these reactions would also be accelerated by the high temperature [40,42]. However, the lack of water would hinder those reactions [38] in the long-term, which could explain the rise of porosity and the fact that the pore network refinement hardly continued at later ages. Moreover, as happened for condition C, in these long-term results for condition D could also have an influence the probably formation of shrinkage cracking [38,56] due to the lack of water.

**Figure 7:**
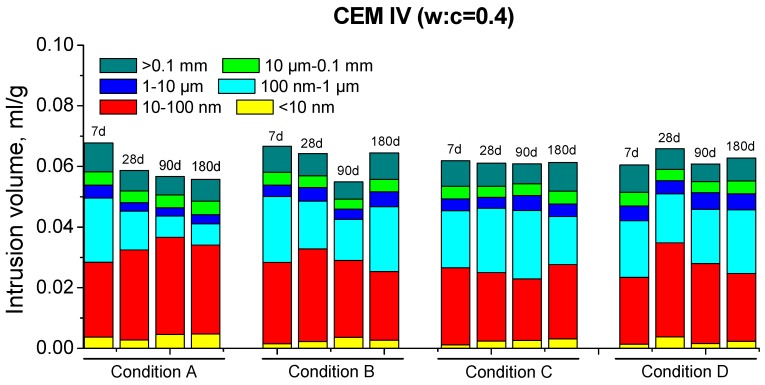
Pore size distributions for CEM IV mortars with w:c ratio 0.4.

**Figure 8:**
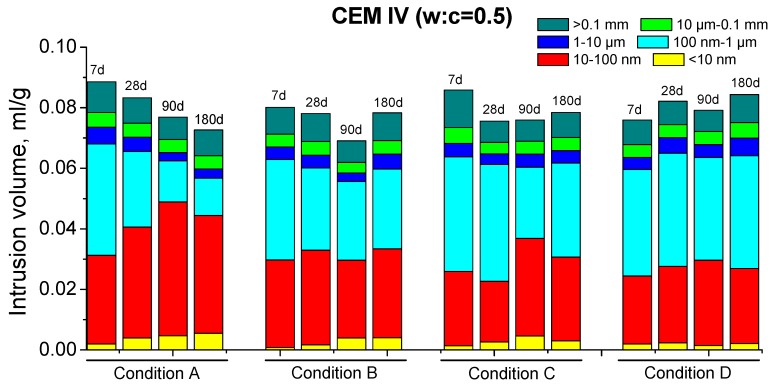
Pore size distributions for CEM IV mortars with w:c ratio 0.5 obtained for each condition.

Regarding the MIP results for CEM IV mortars, the total porosity ones are depicted in Figure 4 and their pore size distributions can be observed in Figure 7 and Figure 8. Firstly, the porosity for condition A decreased until 180 days and the microstructure also became more refined with hardening time. These results coincide with those obtained for CEM II mortars, showing the effect of high RH on hydration and pozzolanic reactions.

The evolution of total porosity and pore size distribution of CEM IV mortars kept in condition B followed a similar tendency than for condition A until 90 days; although, in general, the porous network was less refined for environment B. In the previous discussion of MIP results for CEM II samples (with a lower content of fly ash) exposed to condition B, it was explained that in an environment with a lower temperature, the clinker hydration is slowed down [38], and as a result, the fly ash pozzolanic reactions begin later and they slow down too. This argument could be also valid for CEM IV results.

The relatively low values of total porosity for CEM IV mortars hardened in condition C, and the strong similarity to those obtained for condition A at early ages, could be related to the high enough temperature, as has been already explained for CEM II results. However, in the long-term, the low RH of condition C and the consequent limited availability of water would make the hydration [38] and pozzolanic reactions more difficult. Then, the effects of the solid formation in the microstructure would be produced in a slower way in long times, as suggested by no decrease of total porosity after 7 or 28 days for CEM IV samples with w:c ratio 0.4 and 0.5, respectively. Moreover, this parameter even rose in the long-term, in which could also influence the possible formation of shrinkage microcracks, as has been explained for CEM II samples kept under condition C. Several authors [54,56] have shown that in environments with high temperatures and low RH, shrinkage microcracks could appear in cement-based materials, especially for those with fly ash, in which shrinkage densities several times higher than those noted for OPC ones have been observed.

The tendencies shown by MIP results for CEM IV mortars exposed to condition D were similar than those described for condition C. At 7 days, the total porosities were the lowest of all conditions studied. This could be due to the high temperature, which would speed up the clinker hydration and would advance the starting of pozzolanic reactions, as happened for CEM II mortars. Despite that, after 7 days, the total porosity hardly changed or even increased and the microstructure became less refined. On the one hand, this could probably be related to the lack of water in condition D, which would block the development of hydration and pozzolanic reactions in the long-term, as happened for CEM II samples kept in low RH conditions. On the other hand, the previously mentioned formation of shrinkage microcracks induced by the very low RH could also explain these results.

**Figure 9:**
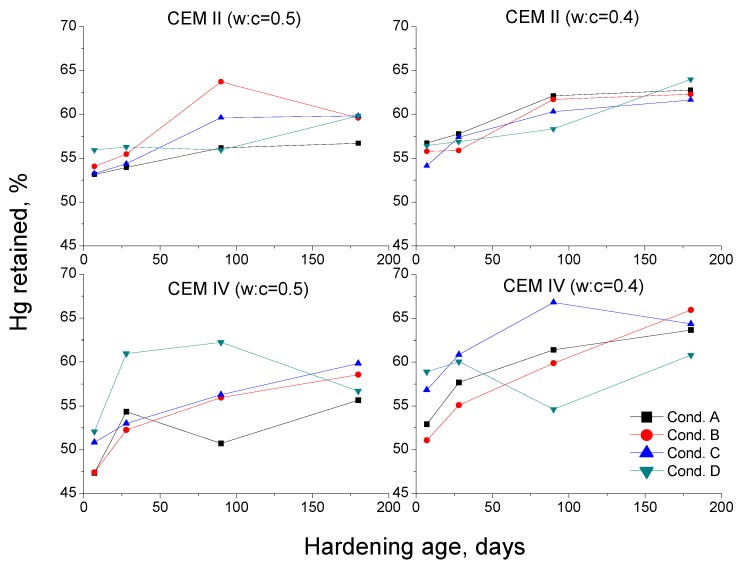
Results of mercury retained after the end of the experiment (MIP) for CEM II and IV mortars.

The Hg retained at the end of the experiment is related to the tortuosity of the pore network of the samples [36]. With regard to this parameter (Figure 9), for the majority of ages and conditions, it was higher for CEM IV than for CEM II mortars. This would reveal a higher tortuosity of the porous network of CEM IV specimens, which is in agreement with the higher microstructure refinement shown by the pore size distributions. The more refined porous network whenever the content of fly ash is increased is a well-known fact, and it has been already demonstrated in many studies [11,57]. In relation to the Hg retained trend observed for CEM IV mortars with w:c ratio 0.5 exposed to condition D, it could be explained in relation to the relatively high temperature and low relative humidity of this condition. The high temperature would accelerate the clinker hydration, and as a consequence, the development of fly ash pozzolanic reactions, which would also start sooner (in the microstructure there would have been enough water available provided from the setting). These reactions produce solid fraction, which increases the tortuosity of the pore network of the material, as would reveal the rise between 7 and 28 days noted for Hg retained. The fact that it did not continue increasing from 28 days could be due to the very low relative humidity in the environment (probably the majority of setting water had already been consumed or removed by drying at that point), which would make more difficult the continuity of clinker hydration and fly ash pozzolanic reactions. Finally, the fall in the long-term noted for Hg retained would be due to the possible shrinkage processes produced by drying, as a consequence of the low environmental relative humidity. Therefore, the formation of shrinkage microcracks would reduce the tortuosity of the pore network of the mortars, lessening the Hg retained, as has been observed, which would coincide with the pore size distributions previously discussed.

### 3.2. Impedance Spectroscopy

Hereafter, the discussion will be focused on impedance spectroscopy parameters results.

Regarding impedance spectroscopy resistances R_1_ and R_2_, they are related to the electrolyte which fills the pores of the sample [36]. Then, in view of MIP results, it would be expected that in the long-term, the highest resistances would be observed for conditions A and B, and the lowest ones, would correspond to conditions C and D.

**Figure 10:**
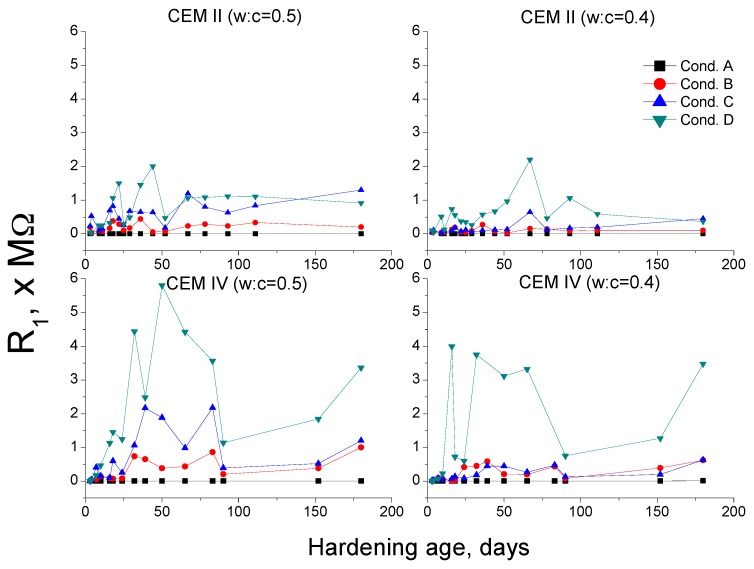
Results of resistance R_1_ for CEM II and IV mortars.

**Figure 11:**
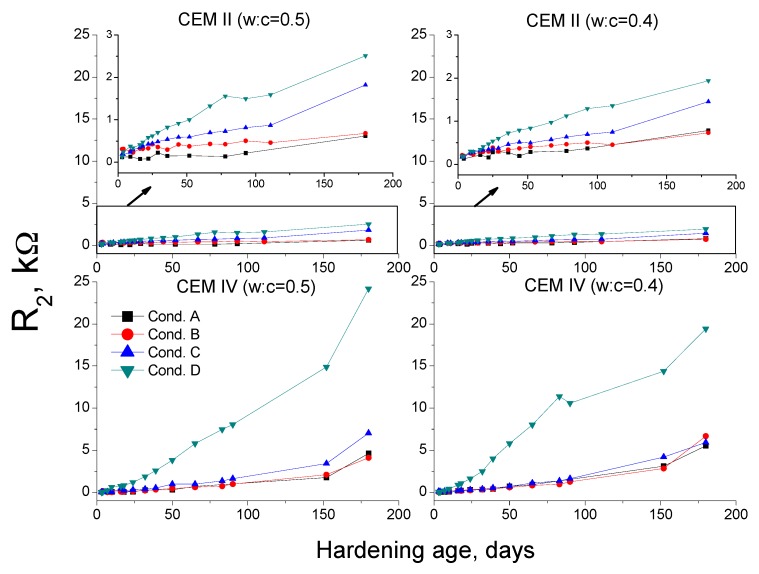
Results of resistance R_2_ for CEM II and IV mortars.

Nevertheless, this did not happen (see Figure 10 and Figure 11) and the condition D showed the highest R_1_ and R_2_ and the condition A the lowest ones. These results agree with those obtained in a previous research [38], in which mortars prepared with OPC and slag cement were exposed to the same environments than those studied in the present research. In that research [38], the resistance behavior was justified due to the drying of samples, which was mostly responsible for the decrease of the volume of pores electrolyte, hiding the effects of the microstructure development. Then, it was concluded that the resistances R_1_ and R_2_ were not a reliable parameter for characterising the changes in the microstructure of cement-based materials in non-saturated samples, like those exposed to non-optimum conditions [38]. This argument would be also valid for impedance resistances results for CEM II and IV mortars obtained now.

**Figure 12:**
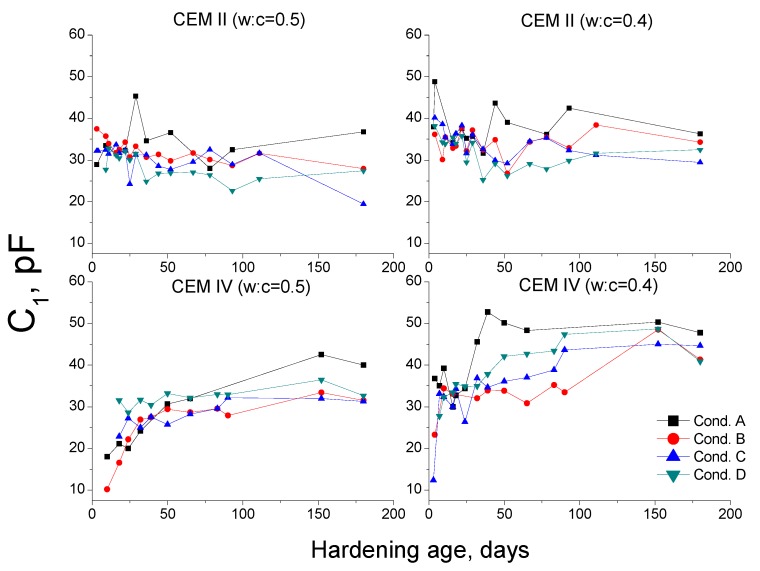
Results of capacitance C_1_ for CEM II and IV mortars.

The capacitance C_1_ is associated to the solid fraction in the samples [36]. Then, it could be expected that this capacitance increases whenever the products of hydration and pozzolanic reactions are formed. The results of capacitance C_1_ are depicted in Figure 12.

In general, for both cements studied, this parameter was higher and its rise with time was more important for specimens exposed to environment A. This would imply that in this condition, there is a faster and more continuous formation of solid phases, which is in agreement with MIP results. This result could be related to the higher RH of condition A, which could facilitate the hydration [38] and pozzolanic reactions [22]. The greater C_1_ value observed for CEM IV mortars at 180 days compared to CEM II ones, especially for w:c ratio 0.4, would be also in keeping with the more refined porous network of CEM IV samples; see Figure 5, Figure 6, Figure 7 and Figure 8.

The increase of capacitance C_1_ for condition B was slower, mainly for CEM IV mortars. However, this parameter rose progressively, and at 180 days, for w:c ratio 0.4 specimens, it reached similar values than those observed for condition A. These results show that the solid phase formation was slower for environment B, which coincides with the MIP results. This behavior could be due to the lower temperature of condition B, which would slow down the development of clinker hydration [38,54], entailing a later beginning of fly ash pozzolanic reactions. Moreover, the lower water available in environment B could also contribute to make more difficult the development of these reactions [38].

For condition C, generally the capacitance C_1_ at early ages was higher and increased faster for both types of cement compared to condition B. These results would mean that in the short-term the formation of solids would be produced relatively quick, which would be also in accordance with MIP results (effect of the relatively high temperature). Nevertheless, in the long-term, the solid phases formation would be produced more slowly, as suggested by the slower rise of capacitance C_1_ observed for condition C compared to condition A. This could be related to the lower RH of condition C, which would make the development of hydration and pozzolanic reactions at greater ages difficult. This result is in agreement with that observed for plain ordinary Portland cement mortars exposed to the same condition [38].

The capacitance C_1_ for mortars exposed to environment D showed relatively high values at early ages. This would indicate that a higher solid formation is produced in the short-term, which would again coincide with MIP results (effect of the high temperature of this condition [38,40,42]). However, the capacitance C_1_ hardly increased in the long-term, as happened with total porosity, probably due to the very low RH in condition D.

**Figure 13:**
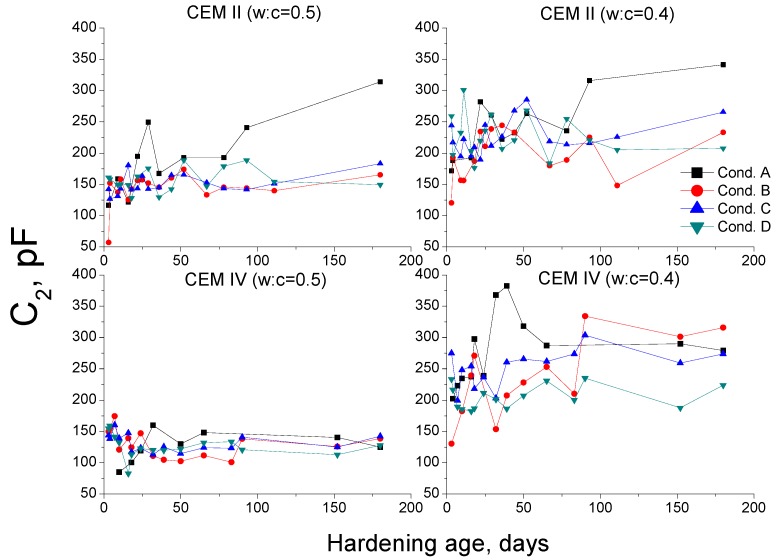
Results of capacitance C_2_ for CEM II and IV mortars.

The capacitance C_2_ is related to the pore surface in contact with electrolyte present in the material and gives information about the formation of CSH gel layers, which occupy the pores [58]. This parameter is very sensitive to the variations of the amount of water in the pores [37,38]. The capacitance C_2_ results are shown in Figure 13.

In general, the highest values and rise with time of capacitance C_2_ have been observed for condition A. Moreover, it continuously increased during the studied period. This result is in accordance with capacitance C_1_ and MIP ones, and it showed the greater formation of solid phases in this condition, which would increase the pore surface.

The initial C_2_ values for condition B were the lowest of all studied environments. However, at later ages, they were similar or even higher than those observed for conditions C and D, but lower than environment A ones. These results would show that the increase of pore surface in condition B, due to solids formation, was produced more slowly, probably by the effect of the lower temperature of this environment, as has been already explained for MIP and capacitance C_1_ results.

The CEM II and IV mortars hardened under condition C had C_2_ values relatively highly compared to the rest of environments. However, the rise with time of this parameter was not as noticeable as observed for condition A. The evolution of capacitance C_2_ in condition C suggests that at early hardening ages, there was a moderately fast formation of solid fraction, which was slowed down in the long-term. Again, this result coincides with previous ones, and it could be related to the low RH in the environment, which would hinder the development of hydration and pozzolanic reactions at greater ages.

With respect to condition D, the capacitance C_2_ also showed high values compared to the rest of conditionsin the short-term. Nevertheless, since then this parameter hardly rose with time. In view of that, it seems that in condition D, new solids would be formed quickly at early ages, which would increase the pore surface, although in the long-term this formation would decrease considerably. In general, C_2_ results are in agreement with capacitance C_1_ and MIP ones, so their discussion is also valid for capacitance C_2_, and they show the effect of combined high temperature and very low RH.

Finally, it is important to emphasize the fact that the capacitance C_2_ for condition D did not change at higher ages, while total porosity increased and capacitance C_1_ decreased. This could confirm the hypothesis of the appearance of shrinkage microcracks in dry conditions. This result has been also observed for plain ordinary Portland cement and slag cement mortars exposed to the same condition D in a previous research [38]. This could be explained because the crack walls formed due to the lack of water would not be filled with electrolytes, so the appearance of shrinkage would not entail a rise of the solid-electrolyte interphase, and thus not contributing to the value of capacitance C_2_ [38]. On the other hand, the shrinkage cracks would suppose a loss of solid fraction, which would bring about a fall of capacitance C_1_ and a rise of total porosity, as has been observed. Then, the present results obtained for fly ash cement mortars, in addition to those previously obtained for OPC and slag cement mortars [38], would strengthen the idea that the combined study of both capacitances C_1_ and C_2_ could be very useful for analyzing the effects of shrinkage in the long-term in cement-based materials hardened in conditions with low RH.

## 4. Conclusions

The main conclusions that can be drawn from the results previously discussed can be summarized as follows:

- The influence of the environmental condition on microstructure of fly ash cement mortars can be studied with impedance spectroscopy, using the equivalent circuits already defined for ordinary Portland cement.
- A high environmental relative humidity entails a more refined microstructure of CEM II and IV mortars, because the high availability of water facilitates the clinker hydration, producing a quicker formation of portlandite, which permits an earlier beginning of fly ash pozzolanic reactions.
- Conditions with high temperature bring a more refined microstructure and a lower porosity of CEM II and IV mortars in the short-term, because the clinker hydration is accelerated, causing the subsequent earlier development of fly ash pozzolanic reactions.
- The impedance spectroscopy resistances R_1_ and R_2_ obtained for fly ash mortars hardened in conditions with relative humidities lower than 100% are more affected by the drying of the materials than by the microstructure development. This result coincided with those obtained in a previous research for OPC and slag cement mortars exposed to the same conditions, so they would confirm that impedance resistances R_1_ and R_2_ are not suitable for following the microstructural evolution of cement-based materials hardened under non-optimum conditions.
- The impedance capacitances C_1_ and C_2_ allow following the microstructure development of fly ash cement mortars, and their results are in accordance with MIP ones for all conditions studied in this research.
- The rise of total porosity and the pore refinement loss observed in the long-term for the mortars exposed to drier conditions could be due to the appearance of shrinkage microcracks as a consequence of the low environmental relative humidity.
- The combined analysis of capacitances C_1_ and C_2_ evolution could justify the abovementioned formation of shrinkage microcracks for CEM II and IV mortars exposed to the drier environments. This result is also in agreement with that obtained in a previous research for OPC and slag cement mortars exposed to the same conditions, which would corroborate the usefulness of both capacitances for studying shrinkage processes in cement-based materials.

## Figures and Tables

**Table 1:** Characteristics of environmental conditions studied.

Condition	Temperature	Relative Humidity	Represented Climate
Condition A	20 °C	100%	Laboratory condition
Condition B	15 °C	85%	Atlantic climate
Condition C	20 °C	65%	Mediterranean climate
Condition D	30 °C	40%	Extreme condition
